# Mitigating the surge of mpox in Africa: capacity building through public outreach, sample and case management training for healthcare workers and veterinarians

**DOI:** 10.11604/pamj.supp.2025.50.1.44476

**Published:** 2025-04-09

**Authors:** Michael Onyebuchi Iroezindu, Abdulwasiu Bolaji Tiamiyu, Zahra Folasade Parker, Danielle Bartolanzo, Edward Abayomi Akinwale, Melanie Diane McCauley, Lateefat Kikeloma Amao, Adeyinka Jeremy Adedeji, Usman Oladipo Adekanye, Fengming Hu, Chinelo Chidiogo Ekweremadu, Odianosen Ehiakhamen, Ikenna Onoh, Oladipo Ogunbode, Afolabi Akinpelu, Adama Ahmad, Adefunke Oladipo-Opashina, Petra Prins, Mihret Amare, Mamadou Bhoye Keita, Kaba Kourouma, Nathan Anelechi Elvis Okeji, Laura Chittenden, Kayvon Modjarrad, Trevor Adam Crowell, Natalie Denise Collins

**Affiliations:** 1 Emerging Infectious Disease Branch, Center for Infectious Disease Research (CIDR), Walter Reed Army Institute of Research, Silver Spring, Maryland, United States,; 2 United States Military HIV Research Program, Center for Infectious Disease Research, Walter Reed Army Institute of Research, Silver Spring, Maryland, United States,; 3 HJF Medical Research International, Abuja, Nigeria,; 4 Walter Reed Army Institute of Research, Abuja, Nigeria,; 5 Henry M. Jackson Foundation for the Advancement of Military Medicine, Bethesda, Maryland, United States,; 6 Nigeria Centre for Disease Control and Prevention, Abuja, Nigeria,; 7 Virology Division, National Veterinary Research Institute, Vom, Plateau State, Nigeria,; 8 Ministry of Defence Health Implementation Programme, Abuja, Nigeria,; 9 Institut National De Sante Publique, Conakry, Guinée

**Keywords:** Epidemic, mpox, public outreach, case management, One Health

## Abstract

The global mpox outbreak and the current surge in Africa underscore the need for innovative epidemic preparedness/response strategies. We describe a comprehensive One Health capacity-building program for mpox response/control for healthcare workers (HCWs) and veterinarians in West Africa. The study design entailed prospective pre- and post-intervention assessments during a train-the-trainer program. Between March and October 2022, four mpox workshops were conducted for HCWs and veterinarians in Nigeria and Guinea. Additionally, two step-down trainings were conducted in Nigeria. Training modules comprised didactic and practical sessions on human and veterinary mpox epidemiology and management. Participants’ knowledge of mpox was assessed with self-administered pre- and post-training questionnaires. A 5-point Likert scale was used to capture participants’ feedback on eight training outcome parameters. Wilcoxon signed-rank test was used to assess differences between pre-training and post-training scores. Of all participants (152), 146 participants (96.1%) completed all training components, including 104 HCWs (71.2%) and 42 veterinarians (28.8%). Participants’ median age (interquartile range, IQR) was 43.0 (36.0-49.0) years, with 99 males (67.8%). Participant scores on mpox knowledge assessments improved from pre-training to post-training (median 55.0% [IQR 45.0-60.0%] to 80.0% [70.0-90.0%], p<0.001). High post-training satisfaction was observed: 83.3% (85/102) strongly agreed that “I am satisfied with the training event,” while 64.1% (66/103) strongly agreed that “training quality and content met my expectations.” Median scores improved during step-down trainings (N=66) from 65.0% (55.0-75.0%) at pre-training to 85.0% (75.0- 90.0%) post-training, p<0.0001. The train-the-trainer program successfully achieved its objectives and could serve as a model for regional/global mpox epidemic preparedness/response.

## Introduction

Mpox, formerly known as monkeypox, is a zoonotic disease caused by the monkeypox virus (MPXV). Following the eradication of smallpox in 1980, MPXV emerged as the orthopoxvirus of greatest relevance to human populations [1]. Mpox is endemic in rainforest regions of Central and West Africa, where most outbreaks have been reported since its initial identification in humans in 1970 in the Democratic Republic of Congo (DRC) [2]. Prior to 2022, confirmed mpox cases were reported by 11 African countries, including DRC and Nigeria [3]. Imported mpox cases reported in Western countries and Asia have been traced back to endemic regions of Africa [2,4]. The animal reservoir of the MPXV is unknown, although close contact with infected wildlife and peri-domestic animals is thought to be the likely source of primary infections in humans [2]. Traditionally, MPXV has been considered to have limited secondary spread through human-to-human transmission [4,5].

Nigeria has had persistent, focal outbreaks of mpox since its first reported case in 1971 [6]. However, from September 2017 to 31 December 2023, confirmed case numbers surged to 1086 in 34 states and the Federal Capital Territory, with 17 deaths [7]. Imported cases of mpox in the United Kingdom [8-12], United States [13,14], Singapore [15], and Israel [16] have been reported in travelers returning from Nigeria between 2018 and 2021. While mpox is not known to be endemic in several African countries [3], shared geography with countries reporting mpox outbreaks, international travel, and fragile surveillance systems create vulnerability for African countries yet to report mpox cases.

The 2022 mpox epidemic in previously non-endemic countries and clustering in specific groups, primarily sexual and gender minorities, challenged the conventional understanding of mpox disease epidemiology and transmission dynamics [2,17]. Despite the lessons learned during the West Africa Ebola outbreak and the COVID-19 pandemic [18], regional healthcare systems were unprepared for the 2022 global mpox outbreak. By July 2022, the World Health Organization (WHO) declared the outbreak a Public Health Emergency of International Concern (PHEIC), underscoring the need for innovative and concerted global health action [19]. From 1st January 2022 to 10th January 2023, 84,471 confirmed cases of mpox and 285 deaths were reported from 110 countries/territories/areas globally [20]. Progress in public health responses alleviated the global health emergency by May 2023 [21]. However, the mpox public health situation in Africa remains concerning, especially in the DRC and Nigeria where unacceptable mpox morbidity and mortality continue to occur [22]. The concerns for regional mpox containment in endemic African countries prompted the organization of a high-level emergency regional meeting in April 2024, which called for a more strategic approach to address significant gaps necessary for effective mpox response efforts [22].

In recognition of chronic deficiencies in African healthcare systems, a team of collaborators from Nigeria, Guinea, and the United States conceived and implemented an mpox capacity-building training program, which commenced shortly before the onset of the 2022 global outbreak. The collaboration included governmental agencies, military healthcare initiatives, and non-governmental organizations from Nigeria, Guinea, and the United States. The initiative provided comprehensive training for Nigerian and Guinean healthcare workers (HCWs) and veterinarians on various aspects of mpox, using a One Health approach. The project’s objectives were five-fold: 1) to build the capacity of HCWs and veterinarians in general outbreak detection and public outreach efforts per international best practices and national reporting guidelines; 2) to enhance their ability to recognize and detect signs and symptoms of mpox; 3) to build their capacity to perform safe sample collection, handling, transport and biosecurity for mpox diagnosis; 4) to improve their knowledge and proficiency in infection prevention and control (IPC); and 5) to develop training capabilities for the transfer of acquired competencies from trained HCWs and veterinarians to their regional counterparts. This article describes the activities of a “train-the-trainer” (TTT) epidemic preparedness and outbreak response capacity-building program designed to help African countries experiencing mpox outbreaks and other at-risk nations with similar infectious disease threats.

## Methods

**Study design/setting/participants:** a prospective pre- and post-intervention assessment was conducted among HCWs and veterinarians in Nigeria and Guinea. In each country, the participants were selected from each of the geopolitical zones/regions. A convenience sampling of key members of the healthcare and veterinary workforces that lead/support emerging infectious disease response was adopted. Training sites were selected for ease of logistical access. In Nigeria, two 5-day trainings were held in Lagos (cohorts A and B) and one 4-day training was held in Abuja (cohort C; shortened due to a national holiday) in March and April 2022. In Guinea, a single 5-day training was conducted in Conakry in October 2022. The training program was initially designed for Nigeria given the country´s important role in global mpox epidemiology. However, based on the lessons learned from the 2022 global outbreak, the partners justified a critical need to roll out the training in an mpox non-endemic West African country. A Francophone country was selected to train an initial set of experts who could potentially cascade to other French-speaking African countries.

**Training materials:** the training materials were developed and delivered by subject matter experts (SMEs), comprising infectious disease physicians, public health physicians, veterinarians, and laboratorians. A catalogue of slide decks, videos, national mpox case management guidelines, laboratory standard operating procedures, and case finding/characterization forms was used in training sessions. Additional materials provided for the practicum portions included the following:

***Personal protective equipment (PPE) supplies:*** surgical masks, N-95 respirator masks, gloves, disposable gowns, head and shoes covers, impermeable aprons, coveralls, goggles, face shields and rubber boots.

***Hand hygiene supplie***s: seventy percent (70%) alcohol-based hand sanitizer, antiseptic wipes, disposable paper towels, glow gel, germ simulation powder, Veronica buckets, water receivers, liquid soap and ultraviolet (UV) torchlight.

***Case simulation supplies:*** medical simulation mannequin (whole body with adjustable body parts), pillow, couch, beddings, pillow slips, duvet and infrared thermometer.

***Sample collection and waste management supplies:*** alcohol swabs, cotton pads, plaster, tourniquet, vacutainer needles and syringes, vacutainer tubes, cryovial tubes, conical tubes, dacron swabs, trays, tube racks, markers, scalpels, triple packing containers/boxes, cold boxes, sharps containers, biohazard bags and ziplock bags.

***Rodent trapping and sample collection supplies:*** Sherman traps, Tomahawk traps, flagging materials, snap traps, baits, leather gloves, markers, Ziplock™ bags, inhalant anesthesia, injectable anesthesia, Pesola scales (50g and 100g), measuring tape, dissecting scissors, forceps, cryovial tubes, cryovial box, cryovial racks, vacutainer tubes, plastic beaker for utensils, stockings, transfer pipette and tips, diaper/bench paper, mammal guide, lighter, data logger, Global Positioning System (GPS) unit, nobutos, Whatman® FTA™ Card, nitrogen dewar, Powered Air Purifying Respirator (PAPR) and field bags. For the Guinea training, all training materials originally developed in English were translated to French. Additionally, indigenous bilingual professional interpreters were contracted. Participants and facilitators were also provided with translation headphones.

**Figure 1:**
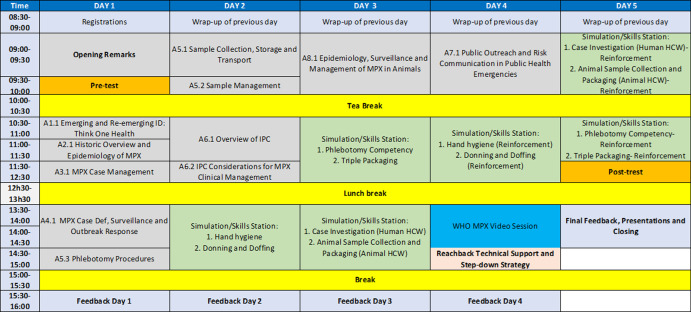
schedule for mpox public outreach and sample collection training in Nigeria and Guinea

**Intervention:** although focused on mpox, the training modules were designed to apply to public health responses for other emerging infectious diseases. The program comprised didactic and practical sessions built into a five-day training schedule (Figure 1). The didactic sessions covered the following: One Health principles, historical overview and epidemiology, case management, case definition, surveillance and outbreak response, phlebotomy procedures, sample collection and management, IPC, public outreach, and risk communication. Various components of IPC were covered including but not limited to chain of infection, universal and transmission-based safety precautions, hand hygiene, general principles of PPE, rational use of PPE, sharps safety, transporting mpox patients, post-mortem care and dignified burial, environmental cleaning, waste management, and mpox vaccination. The practicum sessions focused on hand hygiene, preparation of chlorine (bleach) cleaning solution, donning and doffing of PPE, phlebotomy competency, mpox swab and crust sample collection technique, triple packaging of infectious samples, case investigation, and animal trapping. Question, answer, and feedback sessions were held at the end of each training day. Additionally, a session was dedicated to discussing reach-back and step-down training strategies that leveraged various means to cascade the training to participants´ peers. This session was interactive and highlighted strengths, weaknesses, opportunities, and threats of states/regions and institutions.

**Step-down trainings:** the collaborators conducted two cohorts of 5-day step-down trainings in Lagos, Nigeria between August and September 2022 for HCWs and veterinarians, including some previously trained participants, who anchored the training sessions as trainee-facilitators (otherwise known as mpox champions) under the mentorship of the SMEs. The step-down training agenda and materials were the same as those of the original trainings. The trainees were requested to cascade the training to their peers within four weeks.

**Data management and outcome measures:** a structured questionnaire was used to assess participants´ knowledge of mpox thematic areas before and after the training sessions in each country. The pre- and post-intervention questionnaires were designed as 20 single-answer multiple choice questions drawn from all aspects of the training module. The questionnaires had identical content, were paper-based and were self-administered. The results of these pre- and post-training assessments were entered into a secure access-controlled electronic management system, REDCap. Feedback data were obtained using the Defense Threat Reduction Agency - Biological Threat Reduction Program (DTRA - BTRP) participant feedback form, which comprised eight training outcome parameters: (1) I am satisfied with the training event; (2) training quality, content and presentations met my expectations; (3) instructor was familiar with the topic, and its relevance to my country; (4) instructor´s response to trainee questions was clear and complete; (5) training facility and equipment were appropriate and adequate for the number of students; (6) translation and interpretation was adequate to understand the topic and interact with the instructor; (7) group discussions adequately covered issues relevant to my country; and (8) course material was important and relevant to my current position. Each parameter was scored on a 5-point Likert scale where 5 = strongly agree, 4 = agree, 3 = neutral, 2 = disagree, and 1 = strongly disagree.

**Statistical analysis:** pre-training and post-training questionnaire percentage scores were calculated and summarized with descriptive statistics such as medians, percentiles, and interquartile ranges (IQRs). A Wilcoxon Signed-Rank Test was used to test the difference between the pre-training and post-training scores. The normality assumption for the change in test scores was evaluated by cohort group and occupational category. P-values <0.05 were considered statistically significant. For each training outcome parameter, feedback scores were calculated by dividing the number of responses for each response category by the total number of respondents and multiplying by 100. Analyses were performed using SAS version 9.04.

**Ethical considerations:** ethical approval was not required because this was a public health training activity, supported by national public health authorities. The confidentiality and anonymity of the participants were ensured during pre- and post-training assessments and feedback survey.

## Results

**Demographic characteristics of participants:** the demographic characteristics of the participants are shown in Annex 1. In the Nigeria training, a total of 120 participants (43, 38, and 39 in cohorts A, B, and C, respectively) from 22 Nigerian states were trained. Of the 120 participants, 119 completed all training components, and their demographic information is summarized in Table 1. Seventy-nine (66.4%) trainees were HCWs and 40 (33.6%) were veterinarians. The median (IQR) age of the participants was 43.0 (37.0- 49.0) years, with 80 males (67.2%) and 39 females (32.8%). In the Guinea training, a total of 32 participants from 8 Guinea regions were trained. Of the 32 participants, 27 completed all training components, and their demographic information is summarized in Table 1. Twenty-five (92.6%) trainees were HCWs and 2 (7.4%) were veterinarians. The median age of the participants was 41.0 (35.0 - 47.0) years, with 19 males (70.4%) and 8 females (29.6%). In both the Nigerian and Guinean participants, there was no discrepancy between the pre-training and post-training participants´ demographics, such as age, sex, occupation and professional designation.

**Table 1:** demographic characteristics of participants by country: age, sex, occupational category, and professional designation

Variable	Nigeria cohort; (N=119)				Guinea cohort (N=27)	Combined cohort; (N=146)
	N=119	N=43	N=37	N=39	N=27	N=146
	Total Nigeria median (IQR)	Lagos batch A; median (IQR)	Lagos batch B; median (IQR)	Abuja batch C; median (IQR)	Guinea cohort median (IQR)	Combined median (IQR)
**Age**	43.0 (37.0-49.0)	42.0 (36.0-48.0)	43.0 (36.0-50.0)	45.0 (38.0-51.0)	41.0 (35.0-47.0)	43.0 (36.0-49.0)
	**Total Nigeria; N (%)**	**Lagos batch A; N (%)**	**Lagos batch B; N (%)**	**Abuja batch C; N (%)**	**Guinea cohort; N (%)**	**Combined N (%)**
**Sex**						
Male	80 (67.2%)	30 (69.8%)	20 (54.1%)	30 (76.9%)	19 (70.4%)	99 (67.8%)
Female	39 (32.8%)	13 (30.2%)	17 (45.9%)	9 (23.1%)	8 (29.6%)	47 (32.2%)
	**Total Nigeria; N (%)**	**Lagos batch A; N (%)**	**Lagos batch B; N (%)**	**Abuja batch C; N (%)**	**Guinea cohort; N (%)**	**Combined; N (%)**
**Occupational category**						
Health care workers	79 (66.4%)	26 (60.5%)	19 (51.4%)	34 (87.2%)	25 (92.6%)	104 (71.2%)
Veterinarian	40 (33.6%)	17 (39.5%)	18 (48.6%)	5 (12.8%)	2 (7.4%)	42 (28.8%)
	**Total Nigeria; N (%)**	**Lagos batch A; N (%)**	**Lagos batch B; N (%)**	**Abuja batch C; N (%)**	**Guinea cohort; N (%)**	**Combined; N (%)**
**Profession designation**						
DSNO	39 (32.8%)	14 (32.6%)	10 (27.0%)	15 (38.5)	0 (0.0%)	39 (26.7%)
Epidemiologist	9 (7.6%)	1 (2.3%)	1 (2.7%)	7 (18.0%)	1 (3.7%)	10 (6.8%)
Laboratorian	13 (10.9%)	5 (11.6%)	2 (5.4%)	6 (15.4%)	14 (51.9%)	27 (18.5%)
Doctor/physician	11 (.92%)	3 (7.0%)	5 (13.5%)	3 (7.7%)	5 (18.5%)	16 (11.0%)
Veterinarian	31 (26.1%)	12 (27.9%)	14 (37.8%)	5 (12.8%)	2 (7.4%)	33 (22.6%)
Others^a^	16 (13.4%)	8 (18.6%)	5 (13.5%)	3 (7.7%)	5 (18.5%)	21 (14.4%)

a Other professional designations for Nigeria cohort include: research/data officer (N=6), assistant DSNO (N=3), nurse (N=3), field worker (N=2), biochemist /vaccinologist (N=1), and zoologist (N=1); other professional designations for Guinea cohort include: pharmacist (N=2), not indicated (N=3); IQR: interquartile range

**Figure 2:**
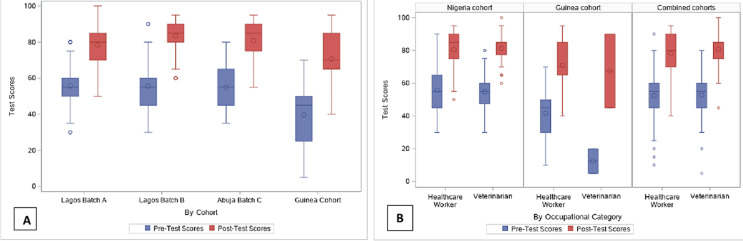
mpox pre- and post-training assessment scores among healthcare workers (HCW) and veterinarians in Nigeria and Guinea, N=146; A) by cohort (Nigeria and Guinea); and B) by occupational

**Pre- and post-training assessments:** the median (IQR) pre-training score vs. post-training score demonstrated an improvement in each batch of the Nigeria cohort as follows: [Lagos batch A] 55.0% (50.0-60.0%) vs. 80.0% (70.0-85.0%), [Lagos batch B] 55.0% (45.0-60.0%) vs. 85.0% (80.0-90.0%), [Abuja batch C] 55.0% (45.0-65.0%) vs. 85.0% (75.0-90.0%). The median (IQR) pre-training score across all Nigeria cohorts was 55.0% (45.0-60.0%), while the median (IQR) post-training score was 85.0% (75.0-90.0%; p<0.0001). The median pre-training score in the Guinea cohort was 45.0% (25.0-50.0%), while the median post-training score was 70.0% (65.0-85.0%; p<0.0001) (Figure 2, panel A). The median score in the combined cohorts (Guinea) improved from 55.0% (45.0-60.0%) at pre-training to 80.0% (70.0-90.0%) at post-training (p<0.0001). As shown in Figure 2 (panel B), among HCWs, median test scores improved as follows: [Nigeria] 55.0% (45.0-65.0%) vs. 85.0% (75.0-90.0%), [Guinea] 45.0% (30.0-50.0%) vs. 70.0% (65.0-85.0%), and [combined] 55.0% (45.0-60.0%) vs. 80.0% (70.0-90.0%). Similarly, veterinarians showed an improved performance as follows: [Nigeria] 55.0% (47.5-60.0%) vs. 85.0% (77.5-85.0%), [Guinea] 12.5% (5.0-20.0%) vs. 67.5% (45.0-90.0%), and [combined] 55.0% (45.0-60.0%) vs. 85.0% (75.0-85.0%).

**Figure 3:**
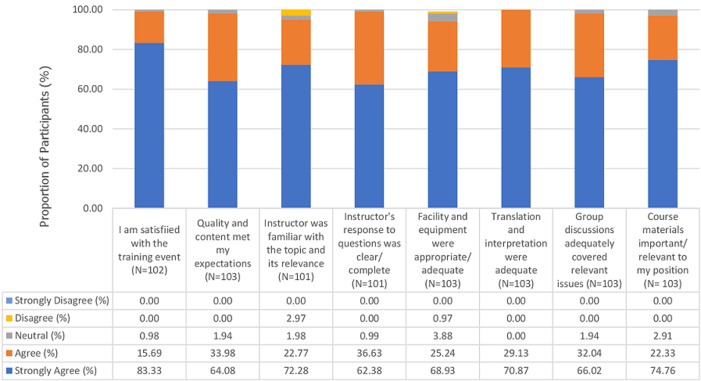
feedback of mpox training participants in Nigeria (n=76) and Guinea (n=27) about the course instruction and quality of training materials, N=103

**Participant feedback:** of 119 Nigeria participants, 76 (63.9%) returned the feedback forms. The response rate was variable across cohorts- 19/43 (44.2%) for cohort A, 19/37 (51.4%) for cohort B, and 38/39 (97.4%) for cohort C. All 27 Guinea participants (100.0%) returned the feedback forms. However, there were minor variations in the total number of participants who provided responses for each of the eight training outcome parameters. Across all cohorts, the survey demonstrated a high level of participant satisfaction with the training across all parameters assessed (Figure 3). Strong agreement (83.3%, 85/102) or agreement (15.7%, 16/102) was expressed for the outcome parameter “I am satisfied with the training event.” Similarly, the majority of participants either strongly agreed (64.1%, 66/103) or agreed (34%, 35/103) that the *“training quality, content and presentations met my expectations.”*

**Step-down training assessments:** sixty-six (66) step-down trainees comprising 51 new participants and 15 previously trained participants (mpox champions) completed the step-down training assessments in two cohorts. Cohort A was made up of 32 participants while cohort B had 34 participants. Of the 66, 48 (72.73%) were HCWs while 18 (27.27%) were veterinarians. For step-down cohort A, the median (IQR) score improved from 65.0% (55-70%) at pre-training to 80.0% (70-85%) at post-training, p<0.0001. The median score for cohort B also improved from pre-training to post-training: 62.5% (55-75%) to 90% (85-90%), p<0.0001. The improvement in median scores remained evident in the combined cohort: 65.0% (55.0-75.0%) at pre-training to 85.0% (75.0- 90.0%) post-training, p<0.0001.

## Discussion

**Meaning of the study:** the overarching goal of the training was to improve the mpox public outreach, sample and case management capacity of HCWs and veterinarians using a One Health approach, while emphasizing the need to cascade the training to their peers at regional and sub-regional levels. This approach empowers the trainees-turned-trainers to gain mastery in the subject, encourages leadership development, and has been effective in other infectious disease train-the-trainer programs [23,24]. As part of the train-the-trainer strategy, trainees were provided appropriate hands-on training materials to expand in-country expertise to (1) enhance regional efforts to combat mpox and other infectious disease threats and (2) provide sustainable mpox training capacity regionally.

Statement of principal findings: the pre- and post-training assessment results of our study suggested significantly improved awareness and knowledge relating to mpox detection and case management in Nigeria and Guinea. Participant knowledge, understanding, and practical skills of IPC measures were improved, including but not limited to donning and doffing of PPE and hand hygiene. Likewise, participants reported enhanced capacity to perform safe mpox sample collection and management. Lastly, the training significantly expanded participants´ knowledge of surveillance, management, and control of mpox in animals. Beyond the statistically significant difference in the pre-training to post-training scores demonstrated in individual and combined country cohorts, the ability of the participants to improve knowledge and skills across various mpox concepts irrespective of their primary discipline, disparity in pre-training mpox public health engagements highlights the applicability of our training model.

**Comparison with previous studies:** the benefits of pre- and post-intervention assessments in capacity building for mpox and other infectious diseases have been previously documented [25-30]. Following a three-day training program conducted in 2011 and 2012 for a total of 126 human HCWs in the DRC, the authors reported significant improvements in HCWs´ knowledge of mpox symptoms, patient care practices and sample collection [25]. Another study demonstrated how a film-based community outreach program can be used to improve the understanding of MPX symptoms, transmission and prevention, by community residents in the Republic of the Congo [26]. Consistent with our findings, a collaborative Ebola training conducted in multiple cohorts using the TTT approach among 14,913 HCWs in 15 Liberian counties showed a remarkable improvement in the knowledge of topics taught at the post-training assessment compared to pre-training scores [27]. Some peculiar strengths of our study in comparison with previous studies are noteworthy, including the multi-country design, the One Health approach, and the deployment of SMEs from an mpox-endemic country to a non-endemic country where the pre-training knowledge of mpox was relatively low. The sample size of our study was slightly higher than the mpox-focused training in the DRC but much lower than the collaborative Ebola-focused capacity-building TTT program in Liberia.

**Strengths of the study:** a One Health framework was adopted to recognize the importance of the interconnection among people, animals, and their shared environment with respect to emerging and re-emerging infectious disease preparedness and response. This multi-agency collaboration not only facilitated cooperation among HCWs and veterinarians from various disciplines for the goal of mpox prevention and control, it also provided an avenue for networking and future collaboration on other shared One Health priorities. Training instructors stressed the need for participants to maintain this framework during their respective regional step-down trainings. The One Health approach has been identified as an important step for addressing global health challenges by other projects [31]. Unfortunately, despite the potential benefits and ongoing advocacy for One Health, palpable challenges to its practical implementation remain such as a lack of national One Health policies, poor coordination of the various sectors, and limited funding [31].

The improvement in mpox knowledge and skills demonstrated during the SME-supervised step-down trainings anchored by previously trained participants in Nigeria lends credence to the success of our TTT program. Additionally, the impact of the program was amplified by the other step-down trainings conducted by the training cohorts in their respective facilities upon their return. Furthermore, trainees have continued to network and support one another in mpox and other emerging infectious diseases public health responses at national and state/regional levels through various communication channels including group mpox social media platforms set up post-trainings. The implementation of our training program during the peak of the 2022 multi-country mpox outbreak made our training program timely and of high global relevance. Poor knowledge and awareness of mpox, especially among HCWs, have been widely reported and remain a significant challenge in global mpox surveillance, prevention and risk mitigation efforts [32-34].

**Unanswered questions and future research:** as already suggested by the WHO, the need to make the most of every opportunity to anticipate, prevent, and control the spread of mpox cannot be overemphasized [35]. So far, there are unanswered questions regarding possible epidemiological and biological factors associated with high mpox mortality in Africa. Given the growing burden of mpox sexual transmission, collaborative research and capacity-building programs on potential mpox and HIV syndemics in Africa are needed. As COVID-19 has taught us, countermeasures alone cannot be the sole solution to addressing mpox outbreaks. National, regional and global public health authorities and other stakeholders must invest heavily in One Health research and other strategies that could potentially improve mpox surveillance, detection, management, prevention, and risk mitigation. This is more critical in Africa where the current mpox surge has partly resulted from knowledge gaps, sub-optimal political will of indigenous governments, and global health inequity in mpox countermeasure deployment [36].

**Limitations:** there were some limitations to the training program. First, while we attempted to include a broad representation of trainees from low- and high-mpox burden states in Nigeria, we found that trainees´ geographical location was prioritized in grouping them per cohort. Fortunately, the lessons learned from Nigeria were used to further improve the criteria for trainee selection, logistics, and training delivery in Guinea. Additionally, trainees shared operational and funding-related challenges in stepping down the training at the regional/sub-regional levels which the collaborators partly mitigated through the SME-supervised step-down trainings in Nigeria. Lastly, there was variability in the interval between the end of the training and the completion of the participant feedback survey. The feedback forms were delivered via e-mail to Nigeria cohort A and B participants within three weeks and one week post-training, respectively. Conversely, Nigeria cohort C and Guinea cohorts completed their forms on-site on the last day of the training. This contributed to lower response rates from cohorts A and B. This difference may have potentially introduced recall bias among participants. Despite the comprehensive strategies we instituted during the Guinea training to prevent a language barrier between the English-speaking facilitators and the French-speaking participants, this challenge might not have been eliminated for all aspects of the program.

## Conclusion

Our mpox capacity building program successfully achieved its training objectives based on the pre- and post-test assessments and feedback survey findings. The synergistic blend of didactic and practicum sessions significantly contributed to the program’s success. The depth and diversity of the facilitators’ expertise ensured broad coverage of the modules. To ensure a far-reaching positive impact of this training model, we recommend cascading the acquired knowledge and skills through step-down trainings led by already trained mpox champions. Finally, global and regional health authorities should consider adopting and adapting a similar training model in their public health response and epidemic preparedness for mpox and other emerging and re-emerging infectious diseases.

## Annexes

Annex 1: state/region of the participants (part of demographic characteristics) (PDF-55.7KB) Annex 2: comparison of the difference between the pre-test and post-test scores of mpox training assessment by country (PDF-126KB)

## References

[1] Jezek Z, Grab B, Paluku KM, Szczeniowski MV (1988). Human monkeypox: disease pattern, incidence and attack rates in a rural area of northern Zaire. Trop Geogr Med.

[2] Bunge EM, Hoet B, Chen L, Lienert F, Weidenthaler H, Baer LR (2022). The changing epidemiology of human monkeypox-A potential threat? A systematic review. PLoS Negl Trop Dis.

[3] Africa Centres for Disease Control and Prevention (CDC) https://africacdc.org/disease/monkeypox/.

[4] Breman JG, Ruti K, Steniowski MV, Zanotto E, Gromyko AI, Arita I (1980). Human monkeypox 1970-1979. Bull World Health Organ.

[5] Fine PE, Jezek Z, Grab B, Dixon H (1988). The transmission potential of monkeypox virus in human populations. Int J Epidemiol.

[6] Alakunle E, Moens U, Nchinda G, Okeke MI (2020). Monkeypox virus in Nigeria: Infection biology, epidemiology, and evolution. Viruses.

[7] Nigeria Centre for Disease Control (NCDC) (2022). An Update of Monkeypox Outbreak in Nigeria: situation report week 52.

[8] Public Health England https://www.gov.uk/government/news/monkeypox-case-confirmed-in-england.

[9] Vaughan A, Aarons E, Astbury J, Balasegaram S, Beadsworth M, Beck CR (2018). Two cases of monkeypox imported to the United Kingdom, September 2018. Euro Surveill.

[10] Vaughan A, Aarons E, Astbury J, Brooks T, Chand M, Flegg P (2020). Human-to-human transmission of monkeypox virus, United Kingdom, October 2018. Emerg Infect Dis.

[11] Hobson G, Adamson J, Adler H, Firth R, Gould S, Houlihan C (2021). Family cluster of three cases of monkeypox imported from Nigeria to the United Kingdom, May 2021. Euro Surveill.

[12] World Health Organization https://www.who.int/emergencies/disease-outbreak-news/item/2022-DON381.

[13] Centers for Disease Control and Prevention https://archive.cdc.gov/www_cdc_gov/media/releases/2021/s0716-confirm-monkeypox.html.

[14] Costello V, Sowash M, Gaur A, Cardis M, Pasieka H, Wortmann G (2022). Imported monkeypox from international traveler, Maryland, USA, 2021. Emerg Infect Dis.

[15] Yong SE, Ng OT, Ho ZJ, Mak TM, Marimuthu K, Vasoo S (2020). Imported monkeypox, Singapore. Emerg Infect Dis.

[16] Erez N, Achdout H, Milrot E, Schwartz Y, Wiener-Well Y, Paran N (2019). Diagnosis of imported monkeypox, Israel, 2018. Emerg Infect Dis.

[17] World Health Organization https://www.who.int/publications/m/item/multi-country-outbreak-of-monkeypox--external-situation-report--1---6-july-2022.

[18] Tusabe F, Tahir IM, Akpa CI, Mtaki V, Baryamujura J, Kamau B (2022). Lessons learned from the Ebola virus disease and COVID-19 preparedness to respond to the human monkeypox virus outbreak in low-and middle-income countries. Infect Drug Resist.

[19] Kozlov M (2022). Monkeypox declared a global emergency: will it help contain the outbreak?. Nature.

[20] Africa Centres for Disease Control and Prevention (CDC) https://africacdc.org/disease-outbreak/outbreak-brief-26-mpox-in-africa-union-member-states/.

[21] Pan African Health Organization (PAHO) https://www.paho.org/en/news/11-5-2023-who-declares-end-mpox-emergency-calls-sustained-efforts-long-term-management-disease.

[22] Africa CDC https://africacdc.org/download/meeting-report-united-in-the-fight-against-mpox-in-africa-high-level-emergency-regional-meeting/.

[23] Tobin CD, Alfred M, Wilson DA, MenkinSmith L, Lehman-Huskamp KL, Schaefer JJ (2020). Train-the-trainer: Pilot trial for ebola virus disease simulation training. Educ Health (Abingdon).

[24] Diesel HJ, Nsagha DS, Sab CM, Taliaferro D, Rosenburg NS (2011). A workshop report on promoting HIV/AIDS understanding through a capacity building train-the-trainer educational intervention. Pan Afr Med J.

[25] Bass J, Tack DM, McCollum AM, Kabamba J, Pakuta E, Malekani J (2013). Enhancing health care worker ability to detect and care for patients with monkeypox in the Democratic Republic of the Congo. Int Health.

[26] Roess AA, Monroe BP, Kinzoni EA, Gallagher S, Ibata SR, Badinga N (2011). Assessing the effectiveness of a community intervention for monkeypox prevention in the Congo basin. PLoS Negl Trop Dis.

[27] Bemah P, Baller A, Cooper C, Massaquoi M, Skrip L, Rude JM (2019). Strengthening healthcare workforce capacity during and post Ebola outbreaks in Liberia: an innovative and effective approach to epidemic preparedness and response. Pan Afr Med J.

[28] Williams VR, Leis JA, Trbovich P, Agnihotri T, Lee W, Joseph B (2019). Improving healthcare worker adherence to the use of transmission-based precautions through application of human factors design: a prospective multi-centre study. J Hosp Infect.

[29] Sakihama T, Honda H, Saint S (2016). Improving healthcare worker hand hygiene adherence before patient contact: A multimodal intervention of hand hygiene practice in three Japanese tertiary care centers. J Hosp Med.

[30] Saint S, Conti A, Bartoloni A, Virgili G, Mannelli F, Fumagalli S (2009). Improving healthcare worker hand hygiene adherence before patient contact: A before-and-after five-unit multimodal intervention in Tuscany. Qual Saf Health Care.

[31] Kelly TR, Machalaba C, Karesh WB, Crook PZ, Glardi K, Nziza J (2020). Implementing one health approaches to confront emerging and re-emerging zoonotic disease threats: lessons from PREDICT. One Health Outlook.

[32] Riccò M, Ferraro P, Camisa V, Satta E, Zaniboni A, Ranzieri S (2022). When a neglected tropical disease goes global: Knowledge, attitudes and practices of Italian physicians towards monkeypox, preliminary results. Trop Med Infect Dis.

[33] Harapan H, Setiawan AM, Yufika A, Anwar S, Wahyuni S, Asrizal FW (2020). Knowledge of human monkeypox viral infection among general practitioners: a cross-sectional study in Indonesia. Pathog Glob Health.

[34] Sallam M, Al-Mahzoum K, Dardas LA, Al-Tammemi AB, Al-Majali L, Al-Naimat H (2022). Knowledge of human monkeypox and its relation to conspiracy beliefs among students in Jordanian health schools: Filling the knowledge gap on emerging Zoonotic Viruses. Medicina (Kaunas).

[35] World Health Organization https://www.who.int/publications/m/item/multi-country-outbreak-of-monkeypox--external-situation-report--8---19-october-2022.

[36] Iroezindu MO, Crowell TA, Ogoina D, Yinka-Ogunleye A (2023). Human mpox in people living with HIV: Epidemiologic and clinical perspectives from Nigeria. AIDS Res Hum Retroviruses.

